# Synthesis of Functionalized Six-Membered-Ring Azahelicenes

**DOI:** 10.3390/molecules27082522

**Published:** 2022-04-14

**Authors:** Francesca Fontana, Benedetta Bertolotti

**Affiliations:** 1 Dipartimento di Ingegneria e Scienze Applicate, Università di Bergamo, 24044 Dalmine, Italy; 2 Dipartimento di Chimica, Materiali e Ingegneria Chimica Giulio Natta, Politecnico di Milano, 20131 Milan, Italy; benedetta.bertolotti@polimi.it

**Keywords:** azahelicenes, photochemical cyclization, cycloisomerization, substitution, azoniahelicenes, helquats

## Abstract

Functionalization, namely the introduction of side groups onto the molecular scaffold of a helicene, may have either the purpose of modifying the electronic properties of the parent helicene, e.g., by adding electron-withdrawing or electron-donating groups, or the scope of providing the helicene with a “handle”, which can be reacted to bind the molecule to another molecule or to a solid structure, such as a carbon or metal surface, or again to allow for complexation of the helicene with metal ions. The possible approaches are two-fold: the synthesis of the helicene can be performed using starting materials that already contain a side group, or the side group can be introduced after the synthesis of the parent helicene. As azahelicenes are helicenes bearing one or more nitrogen atom(s) in the molecular framework, parent azahelicenes can be functionalized on carbon atoms by exploiting the presence of the electron-withdrawing nitrogen atom. Moreover, they can be transformed into quaternary salts, whose properties are quite different from those of the parent azahelicenes in terms of the solubility and electronic properties. This review aims to provide a survey of the different synthetic methods available to attain this fascinating class of compounds.

## 1. Introduction

Azahelicenes [1,2] constitute a class of heteroaromatic molecules endowed with an extended conjugated system, which imparts them large conductivity and polarizability, exploitable in fields such as optoelectronics [3], catalysis [4], sensors [5], and more. Their intrinsic chirality due to helicity is reflected in their extremely high optical rotation. Their racemization times are shorter than those observed for carbohelicenes of the same structure, and depend on the position of nitrogen onto the molecular framework [6]. To be configurationally stable at room temperature, six-membered ring azahelicenes must possess at least six fused rings, unless appropriately bulky substituents on the terminal rings prevent racemization.

Azahelicenes can furthermore aggregate into relatively stable supramolecular structures (π-stacking, polar interactions); in addition, they exhibit a remarkably long triplet state lifetime [7]. Due to the presence of the nitrogen atoms, these compounds are more polar than carbohelicenes and exhibit basic properties [8,9]. Obtaining functionalized azahelicenes, on the one hand, provides a way to modulate their electronic properties by inserting electron-withdrawing or electron-donating groups; moreover, an appropriately functionalized moiety covalently bonded to the helical scaffold may be used to attach the molecule to a surface of some kind, such as carbon or metal, or to connect it to other molecules or to metal ions. In the literature, there are relatively few instances of functionalized six-membered ring azahelicenes, either obtained by incorporating the functional group in the precursors to the synthesis of the azahelicene, or by inserting it onto the previously prepared azahelicene molecule.

In this review, which is focused on synthetic aspects, only azahelicenes where nitrogen atoms are part of a six-membered ring (azines) are considered; functionalization refers to the introduction, on any part of the structure, of substituents including alkyl groups, aromatics, functional groups of any kind. The synthetic strategies are grouped into (i) methods of direct synthesis, starting from precursors already bearing the desired substituent, and (ii) methods for the functionalization of pre-formed azahelicenes; these two sections deal with substitution onto a carbon atom of the framework. Furthermore, a third section is devoted to quaternarization of the nitrogen atom(s), which allows for obtaining quaternary azahelicenium salts, helquats, and azoniahelicenes.

## 2. Direct Synthesis of Functionalized Azahelicenes

The synthesis of helicenes can be obtained following different strategies, namely (a) oxidative photocyclization, (b) Diels–Alder reactions, (c) Friedel–Crafts reactions, and (d) metal-mediated reactions. Strategies (a) and (d) have been mainly used to obtain the direct synthesis of functionalized azahelicenes by using appropriate starting materials, bearing functional side substituents compatible with the reaction to be performed.

### 2.1. Diarylethene Cyclization

In this approach, an appropriate diarylethene derivative is prepared, which is then cyclized, usually by photochemical methods, to obtain helicene; the diarylethene is generally obtained either by the Wittig reaction between a phosphonium salt and an aldehyde, or by Heck coupling through a vinyl derivative and an aryl halide. It is possible to devise the synthesis by incorporating starting materials containing the desired side groups.

**Scheme 1:**
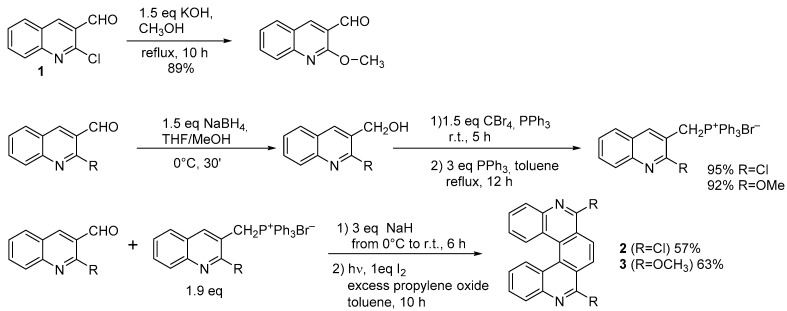
Synthesis of 6,9-dichloro- and 6,9-dimethoxy-5,10-diaza[5]helicene.

The Wittig reaction is compatible with several functional groups, including multiple bonds, halogens, nitro, ether, ester, and acetal groups, which therefore may be present on the aldehyde moiety. The phosphorus ylide may contain multiple bonds and several functional groups; if it contains electron-withdrawing groups, it becomes more stable. This approach was adopted in many cases for carbohelicenes, for instance in [10], where 4-bromobenzaldehyde reacted with the appropriate four-ringed phosphonium ylide to obtain the diarylethene precursor of 2-Br-hexahelicene. For azahelicenes, the only additional limitation concerns the availability of the required commercial starting materials. One reported instance starts from 2-chloroquinoline-3-aldehyde (**1**) to obtain the symmetrical 6,9-dichloro-(**2**) and 6,9-dimethoxy-5,10-diaza[5]helicene (**3**) [11]. Both terms employed in the Wittig condensation originate from the same substituted quinolinaldehyde (Scheme 1); (**1**) is easily accessible by reacting acetanilide with POCl_3_ in DMF at 80 °C [12]. By using differently substituted moieties, it is also possible to obtain the unsymmetrically substituted product.

**Scheme 2:**
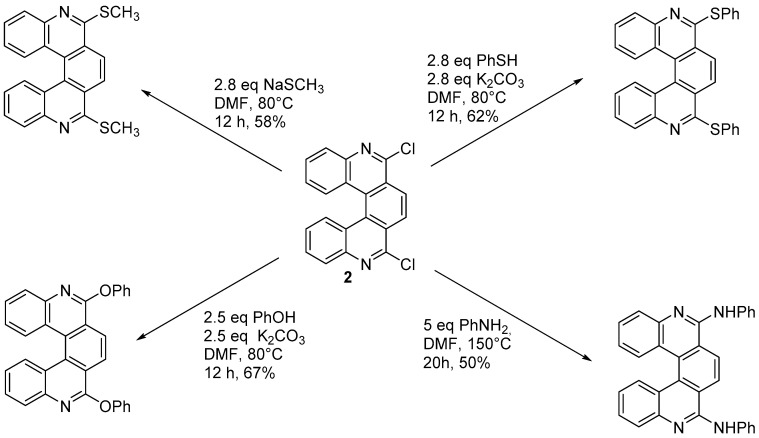
Introduction of functional groups starting from dichlorodiazahelicene **2**.

Starting from 6,9-dichloro-5,10-diaza[5]helicene (**2**) it is possible to substitute chlorine atoms with several functional groups, as depicted in Scheme 2.

The substitution of chlorine atoms with chiral amines provides a way to separate diastereomers, in order to achieve an enantiomeric resolution. However, the five-ringed helicenic scaffold is readily racemized, and the presence of nitrogen substitution accelerates racemization compared to the parent compound [11].

**Scheme 3:**
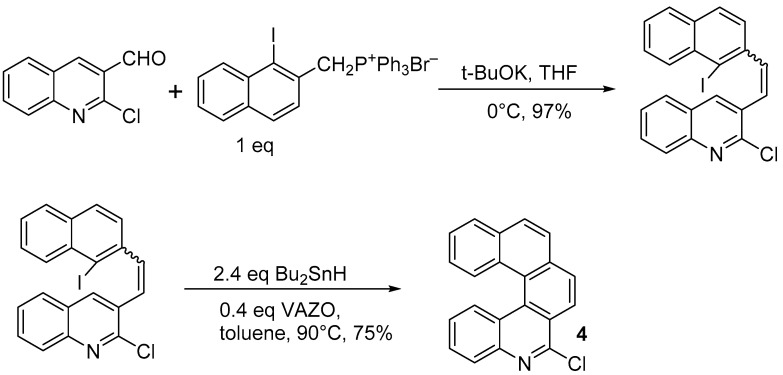
Synthesis of 6-chloro-5-aza[5]helicene **4** by radical cyclization.

In a modification of this approach, Harrowven and coworkers [13] prepared 6-chloro-5-aza[5]helicene (**4**) again by using (**1**) as the starting material, and reacting it with a phosphonium salt bearing an iodine atom, to be used for a non-photochemical radical coupling reaction (Scheme 3).

**Scheme 4:**
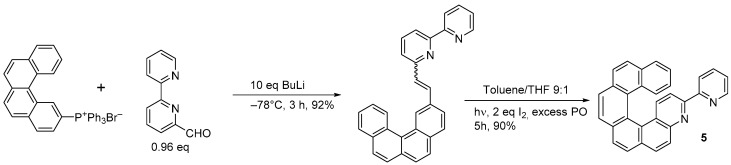
Synthesis of racemic 3-(2-pyridyl)-4-aza[6]helicene (**5**).

3-bromo-4-aza[6]helicene was also prepared following the Wittig condensation. The photochemical ring closure strategy [14] started from 6-bromo-2-pyridinecarboxaldehyde, which was condensed with benzo[g]phenanthren-3-ylmethyl triphenyl phosphonium bromide through the use of BuLi in THF to obtain the corresponding diarylethene in a 90% yield. This was then irradiated with visible light in toluene with I_2_ (2 equiv.) and excess propylene oxide (PO) for 15 h to yield 3-bromo-4-aza[6]helicene in a 45% yield. In the same way, racemic 3-(2-pyridyl)-4-aza[6]helicene **5** was synthesized in two steps from 2,2′-bipyridine-6-carbaldehyde by the Wittig reaction with (benzo[*g*]phenanthren-3-ylmethyl)triphenyl phosphonium bromide followed by a photocyclization reaction (83% overall yield, Scheme 4) [15], and 3-(2-pyridyl)-4-aza[4]helicene was prepared by using (2-methylnaphthyl) triphenyl phosphonium bromide, with an overall yield of 61%.

These compounds were used to realize Pt complexes acting as chiroptical switches. The 2,2′-bipyridine unit is endowed with remarkable stability and exceptional coordination chemistry. Functionalized helical bipyridine systems exhibit, in addition to the usual azahelicene properties, a rich and high-affinity coordination chemistry with metals, a good acid/base and alkylation reactivity, and redox activity.

**Scheme 5:**
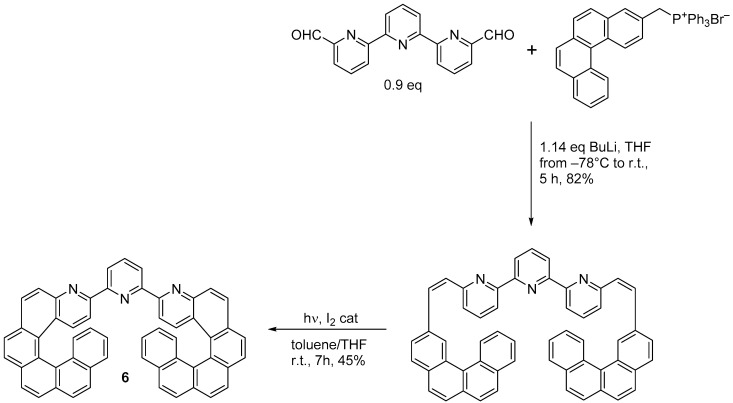
Synthesis of 2,6-di[3-(4-aza[6]helicenyl)]pyridine (**6**).

A chiroptical switch based on reversible Zn(II) complexation by the double azahelicene (**6**) was synthesized by the same research group through double photochemical cyclization (Scheme 5) [16].

**Scheme 6:**
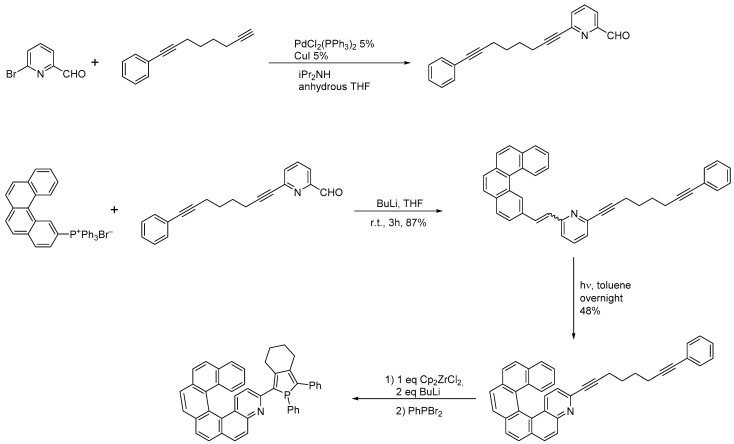
Synthesis of a 4-aza[6]helicene bearing a phosphole moiety in position 3.

The same group also synthesized a three-substituted 4-aza[6]helicene by preparing a diarylethene already bearing a dialkynyl substituent, which, after cyclization, was then reacted further to obtain a phosphole-substituted azahelicene, as reported in Scheme 6 [17].

**Scheme 7:**
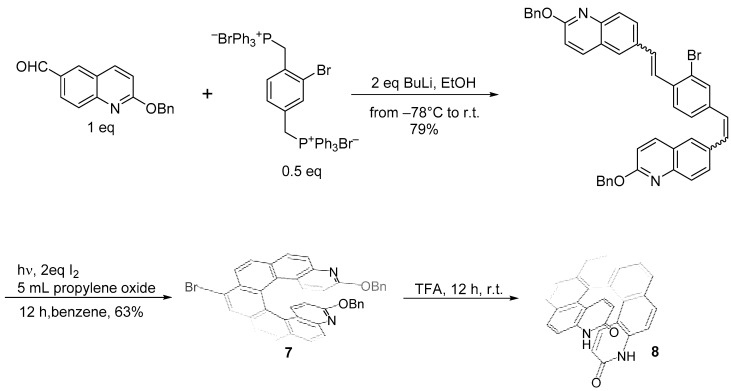
Synthesis of substituted 4,15-diaza[7]helicene **7**.

A Br-substituted diaza[7]helicene (**7**) was prepared [18] through the Wittig condensation, as shown in Scheme 7.

Removal of the benzyloxy groups from (**7**) by TFA yields the corresponding brominated pyridinone **8** in a 63% yield. The bromine atom can be substituted with the COCH_3_ group. Pd- and CuI-cocatalyzed coupling of TMS-acetylene with **7** yielded the corresponding ethynyl helicene, which, by treatment with trifluoroacetic acid, cleanly liberated the pyridinone hydrogen bond sites and simultaneously deprotected the TMS-acetylene moiety, generating the methyl ketone derivative analogous to **8** in a nearly quantitative yield.

**Scheme 8:**
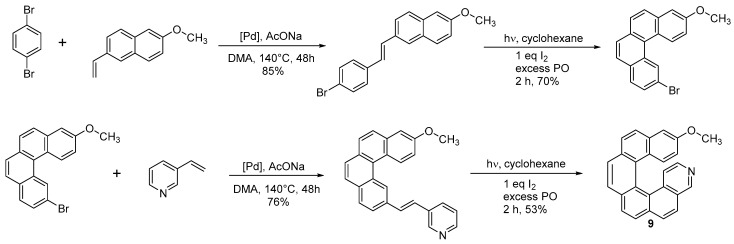
Synthesis of 14-methoxy-3-aza[6]helicene **9**.

The same approach held for the Heck coupling, where both the alkene and the halogen-bearing moieties used to obtain the diarylethene could bear substituents. 14-methoxy-3-aza[6]helicene (**9**) was synthesized through a double Heck condensation-photochemical oxidation approach (Scheme 8) [19].

The Heck couplings were obtained by using excess of the commercially available vinylderivatives in the presence of 3 equiv. AcONa and 1% Hermann’s palladacycle [trans-di(μ-acetato)-bis[o-(di-o-tolylphosphino)-benzyl]dipalladium] as the catalyst in DMA. The -OCH_3_ group can be easily transformed in -OH by treatment with a BBr_3_ solution in CH_2_Cl_2_ at RT for 3 h, with an excellent yield. The hydroxy derivative could serve as N–O bidentate ligands in asymmetric synthesis or as building blocks for supramolecular architecture. Although not many instances have been described in the literature, this approach is quite general.

**Scheme 9:**
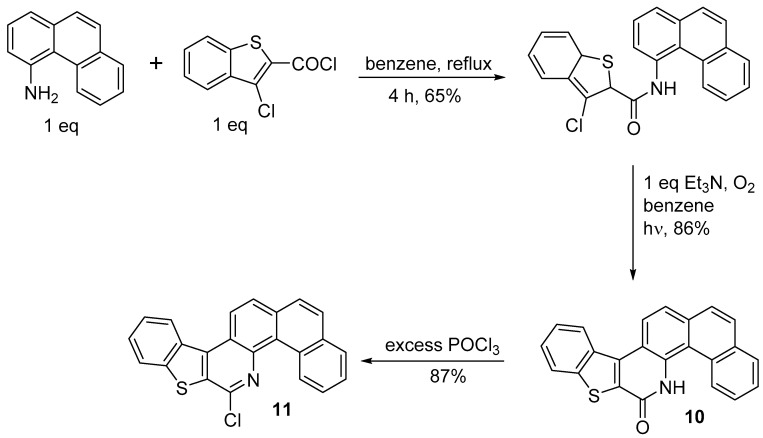
Synthesis of 8-chlorobenzo[4,5]thieno[2,3-c]naphtho[2,1-h]quinoline **11**.

Only two successful syntheses of stilbenoid precursors by the photocyclization of amides have been described [20]. In the first, photochemical ring closure of the amide provides lactam **10**, which is then converted to double helicene **11** by POCl_3_ (Scheme 9).

**Figure 1:**
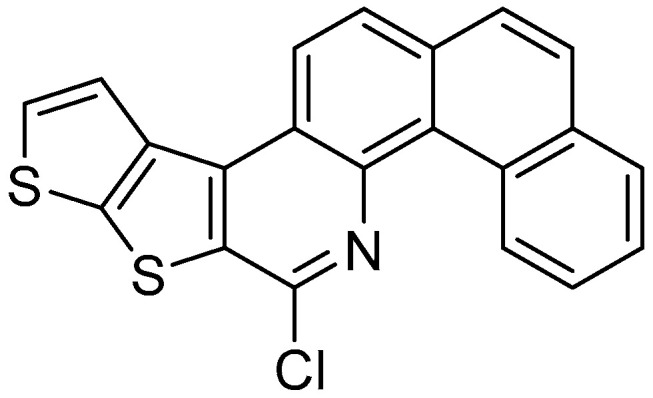
8-chloronaphtho[2,1-h]thieno[3’,2’:4,5]thieno[2,3-c]quinoline.

In the second, the same research group obtained the dithienocondensated aza[4]helicene (Figure 1) by the same procedure; however, the yield of the last step with POCl_3_ was moderate (39%).

### 2.2. [2 + 2 + 2] Cycloisomerization

**Figure 2:**
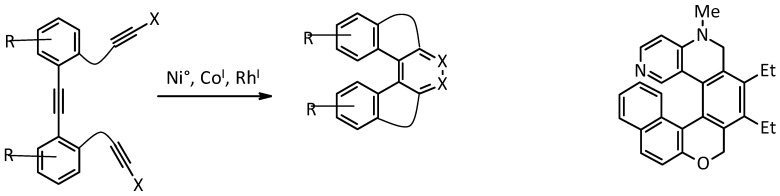
Scheme of the general [2 + 2 + 2] cycloisomerization reaction (**left**); a nitrogen-containing helicenoid structure obtained in this way (**right**).

This approach to the synthesis of helicenes involves intramolecular [2 + 2 + 2] alkyne cycloisomerization catalyzed by Co^I^, Ni^0^, or Rh^I^ to provide the simultaneous formation of three or more cycles in a single step, giving access directly to the desired helicene, or to its tetrahydro derivative, to be oxidised in a subsequent step. This strategy has been extensively used for the synthesis of electron-rich systems, namely carbo- and oxa-helicenes [21], while only limited examples are reported for electron-poor pyridine moieties (Figure 2).

**Scheme 10:**
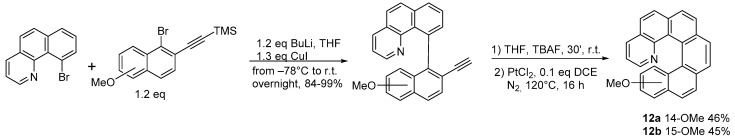
Synthesis of methoxy-substituted 1-aza[6]helicenes.

Most frequently, unsubstituted azahelicenes are prepared by this method [22]. Some attempts to apply this strategy to alkyl-substituted azahelicenes were only partially successful. Cycloisomerization of an azabiphenylylnaphthalene catalyzed by InCl_3_ and PtCl_4_ allowed for the synthesis of 6,10-dimethyl-2-aza-[6]helicene in an 80% yield [23], while the same reaction, applied to the synthesis of 6,10-dimethyl-2,14-diaza-[6]helicene, failed despite the screening of a number of different catalyzers. In another instance [24], diversely substituted 1-aza[6]helicenes are produced in gram scale, starting from methoxy-substituted azahelicene (**12**), obtained as in Scheme 10.

**Scheme 11:**
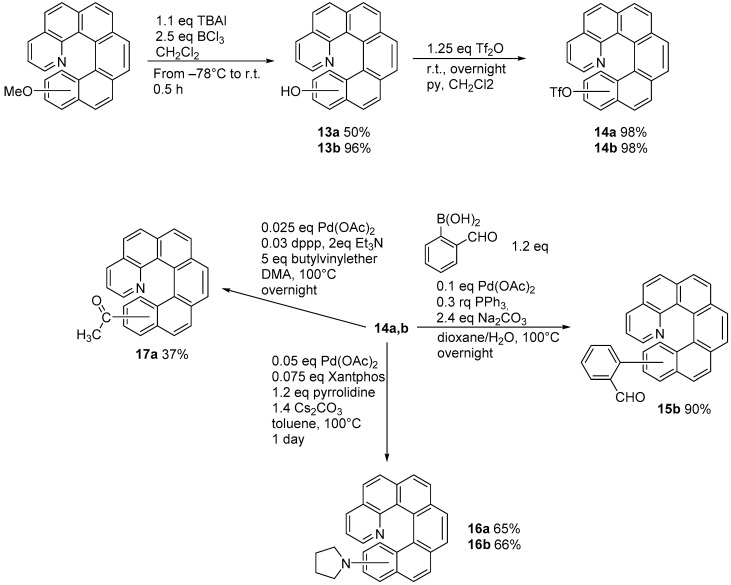
Transformations of functional groups onto the 1-aza[6]helicene framework.

The methoxy group, which can be located either in position 14 (**12a**) or 15 (**12b**), can then be converted relatively easily and with fair to good yields into different functional groups (Scheme 11). Furthermore, the precursors to compounds **12** show atropoisomeric chirality and could be resolved into stable enantiomers, thus enabling the obtainment of compounds **12a**,**b** with an enantiomeric excess of more than 90%.

**Scheme 12:**
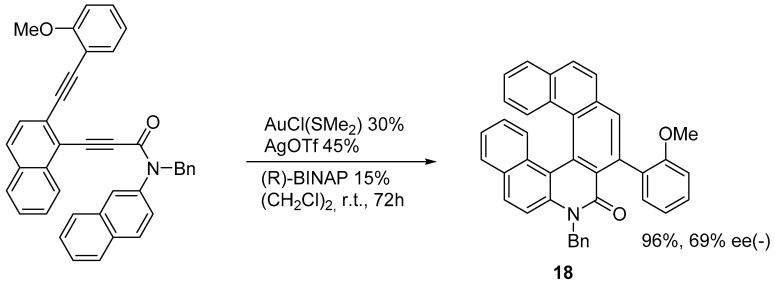
Preparation of a substituted 7-aza[6]helicenone.

The enantioselective synthesis of azahelicenes and S-shaped double azahelicenes has been achieved via the Au-catalyzed sequential intramolecular hydroarylation of alkynes [25]. By using the catalytic system indicated in Scheme 12, the azahelicenone **18** is obtained in very good yield with 69% ee starting from an achiral precursor. It is also possible to reduce the amount of the catalyzers by operating at 80 °C: in this case, although, the yield decreases to 80%, and the ee decreases to 56%. When a phenyl group is introduced as a substituent in place of 2-methoxyphenyl, both the yield and ee decrease substantially.

Following the same procedure, products analogous to 18 can be prepared, in which one or both terminal aromatic rings bear two methoxy groups in positions 1,3.

**Figure 3:**
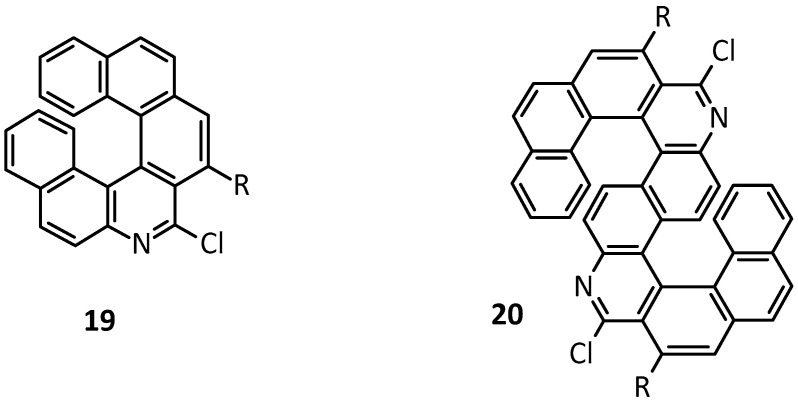
Azahelicenes obtained by sequential intramolecular Au-catalyzed diyne or tetrayne hydroarylation.

Starting from a product analogous to **18** in which the nitrogen atom bears a 4-methoxybenzyl group, treatment with CF_3_COOH followed by reaction with POCl_3_ and PhNMe_2_ (-)-8-chloro-2-(2-methoxyphenyl)-7-aza[6]helicene **19** (Figure 3) obtained a 67% yield, with 74% ee. By using the same reaction strategy, it is also possible to obtain the S-shaped double azahelicene **20** represented in Figure 3, with >99% ee of the (+) enantiomer.

Double azahelicenes showed red shifts of absorption and emission maxima and higher quantum yields in the CHCl_3_ solution compared with azahelicenes. The optical rotation values of double azahelicenes were smaller than those of the corresponding single azahelicenes.

### 2.3. Other Synthetic Methods

**Scheme 13:**
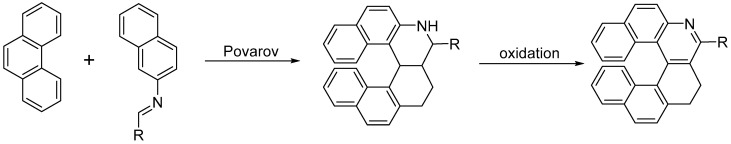
The Povarov approach to substituted azahelicenes.

The Povarov reaction is a formal inverse-electron demand [4 + 2] cycloaddition between electron rich-alkenes, acting as dienophiles, and *N*-alkylidene anilines acting as electron-poor dienes. The primary Povarov cycloadduct is a tetrahydroquinoline that can be oxidized to the corresponding quinoline; therefore, it can be used to grant easy access to helical-shaped compounds (Scheme 13) [26].

**Scheme 14:**
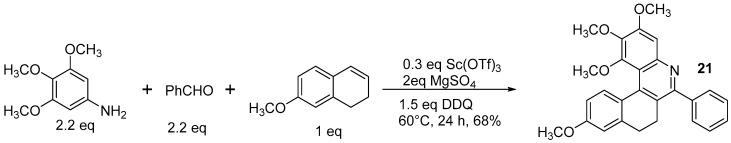
The formation of dihydroazahelicene **21** by the Povarov reaction.

Actually, the reaction depicted in Scheme 14 brings about the formation of the dihydroderivative **21**, which cannot be further oxidized even in much harsher conditions (5 equiv. of DDQ, DMF, microwave 150 °C, 12 h).

**Scheme 15:**
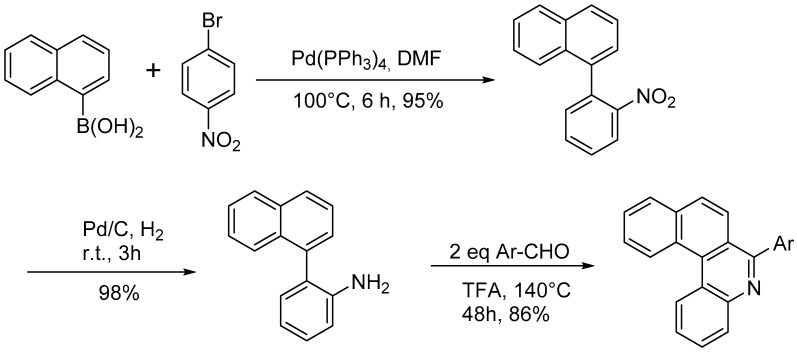
Synthesis of aryl-substituted aza[4]helicenes by Pictet-Spengler reaction.

The Pictet-Spengler reaction was used to prepare a functionalized aza[4]helicene as depicted in Scheme 15 [27].

The reaction can be performed by both electron-deficient and electron-rich aldehydes, resulting in an aryl substituent that can bear groups such as –OCH_3_ or –NO_2_. In this paper, the optical properties of the aza[4]helicenes were studied by UV spectroscopy, finding absorption bands in CH_2_Cl_2_ with maxima at 274–307 nm, with the presence of electron-withdrawing groups resulting in absorption at longer wavelengths than the electron-donating groups; the positions of the substituents had no obvious effect on the maximum of the absorption peak. In the fluorescent spectra, the emission maxima for the helicene skeleton were observed at about 400 nm, and the substituent groups and their positions had no obvious effect on the emission, while the fluorescence intensities were weak. The methoxy group onto the aromatic ring can be demethylated with 98% yield by BBr_3_ in dichloromethane. This method could also be applied to azahelicenes with more than four rings.

**Scheme 16:**
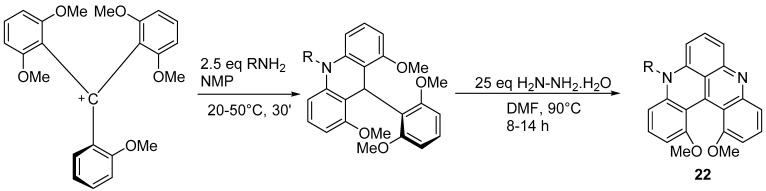
Synthesis of a configurationally stable substituted diaza[4]helicene.

The configurationally stable diaza[4]helicene **22** is prepared starting from the tris(2,6-dimethoxyphenyl)methyl cation following the strategy indicated in Scheme 16 [28].

R can be alkyl, phenyl, benzyl, -NH_2_, -CH_2_CH_2_OH, or -CH_2_CH_2_ NH_2_. Yields range from 80 to 97%. This synthetic procedure is general, modular, and highly tolerant to functional groups. Single enantiomers of the [4]helicenes were obtained by chromatographic resolution, with a high racemization barrier. These helical quinacridines are effective dyes showing interesting absorption and emission properties, that can be modulated as a function of pH. Their fluorescence is weak and they show relatively modest changes in emissive properties upon protonation.

**Scheme 17:**
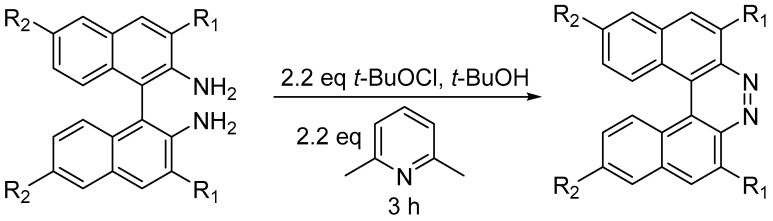
Synthesis of substituted 7,8-diaza[5]helicenes.

Substituted 7,8-diaza[5]helicenes were prepared by oxidative ring closure of 1,1′-binaphthalene-2,2′-diamines (BINAMs, Scheme 17) [29].

t-BuOCl acts as bland oxidizing agent, while 2,6-lutidine serves as weak base to neutralize HCl generated in the process. The method is moderately tolerant to functional groups: R_1_ can be H, CH_3_, Br, Ph, and COOCH_3_, while R_2_ can be H, Br, or butyl. The reaction takes place at RT in 3 h with yields ranging between 44 and 91% [29]. For these diazahelicenes, UV–VIS absorption spectra were recorded, observing a red shift over the whole region upon the introduction of methyl or phenyl groups in the 3,3′ positions, while on the opposite side the introduction of 6,6′-*n*-Bu substituents caused a blue-shift of n–π * transitions (380–430 nm), while π–π * transitions (300–340 nm) were red-shifted. Cyclic voltammetry experiments were used to estimate the LUMO energies, which ranged from −2.92 to −3.13 eV.

**Scheme 18:**
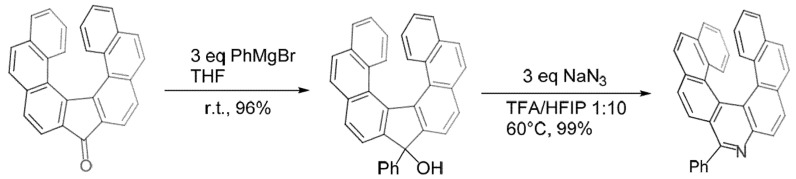
Synthesis of aryl-substituted aza[7]helicenes by ring expansion.

A very recent paper [30] reported the synthesis of a substituted aza[7]helicene by ring expansion starting from a helical ketone, which was reacted with a carbanion to obtain a five-membered ring alcohol. This latter then undergoes ring expansion by reaction with NaN_3_, with subsequent rearomatization/deprotonation to yield an aryl-substituted aza[7]helicene (Scheme 18).

A variety of mono- and poly-cyclic aryl moieties were attached to the aza[7]helicene with excellent yields through the use of an appropriate Grignard reagent; both electron-withdrawing and electron-donating substituents on the aryl ring were tolerated and even aliphatic chains could be introduced. The enantiomers were separated by chiral HPLC. Under the irradiation of UV light (365 nm), some of these compounds, notably those bearing Ph, *n*-Bu,*p*-OPh, and 7-(tert-butyl)pyren-2-yl, displayed a bright green luminescence. The quantum efficiency of the n-Bu-substituted derivative leaps to 18% in comparison to ∼7% for its aryl analogues. The longest absorption maximum in UV−VIS spectra is at ∼433 nm.

## 3. Attaching Substituents to Azahelicenes

This approach is based on building the unsubstituted azahelicene framework, to be then reacted by exploiting the presence of the nitrogen atom for directing the substitution.

### 3.1. Reactions of N-Oxides

**Scheme 19:**
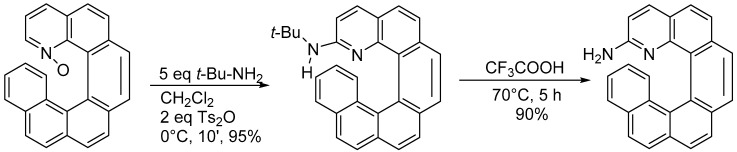
Exploiting the N-oxide functionality to introduce a vicinal amino group.

Carbohelicenes can be functionalized by means of the Friedel–Crafts reaction, and it was demonstrated [31] that electrophilic aromatic substitution is mainly directed in the five-position, and to a minor amount towards the seven-position. In this way, acylation, halogenation, and nitration can be obtained. However, six-membered ring azahelicenes are electron-poor aromatic compounds and therefore they are reactive towards nucleophilic reagents. One approach to the functionalization of azahelicenes can be the oxidation of the nitrogen atom by *meta*–chloroperbenzoic acid to N-oxide, followed by nucleophilic addition and elimination, to introduce amino substituents in the vicinal position [32]. In this way, enantiomerically pure (M)-1-aza[6]helicene N-oxide was reacted with 5 equiv. of an amine R-NH_2_ in CH_2_Cl_2_, upon the addition of 2 equiv. of Ts_2_O at 0 °C, to yield 2-RNH-1-aza[6]helicene. This latter is then treated with TFA at 70 °C during 5 h to yield 2-amino-1-aza[6]helicene 23 in very good yield (Scheme 19).

The same approach was adopted for benzocondensated 1-aza[6]helicene [33].

**Scheme 20:**
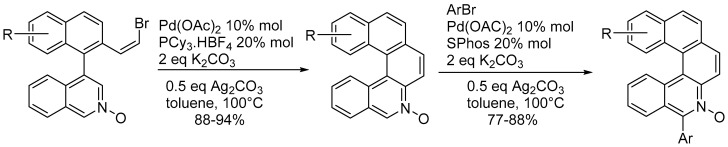
Synthesis and arylation of 6-aza[5]helicene N-oxide.

Another research group described the synthesis and arylation of the N-oxide through the use of Pd catalysis [34]. N-oxide was prepared from a precursor already bearing the N-oxide functionality, it then underwent direct arylation in the vicinal position by reacting it with ArBr (Scheme 20).

Both the helicene precursor and the aryl group may bear substituents; the azahelicene can bear a methoxy group in positions 2, 12, or 13, or a CF_3_ group in position 2. The aryl group in the ArBr moiety can be 4-methoxy, 4-methyl, 4-CF_3_, or 4-CHO. With an appropriate choice of the ligand, namely SPhos (2-bicyclohexylphosphino-2*’*,6*’*-dimethoxybiphenyl) instead of the triphosphinic ligand PCy_3_.HBF_4_, it was possible to obtain the arylated azahelicene-N-oxide in a one-pot procedure with a 56% yield.

### 3.2. Substitution Reactions

Six-membered-ring azahelicenes are extensively conjugated azines; as such, they show the reactivity of azines, with electron-poor aromatic rings prone to nucleophilic aromatic substitution in the ortho- and para-positions with respect to the nitrogen atom. Usually, monoazahelicenes only have one reactive ortho-position, unless the nitrogen atom is located on the terminal ring, in which case it has two non-equivalent activated positions.

**Scheme 21:**
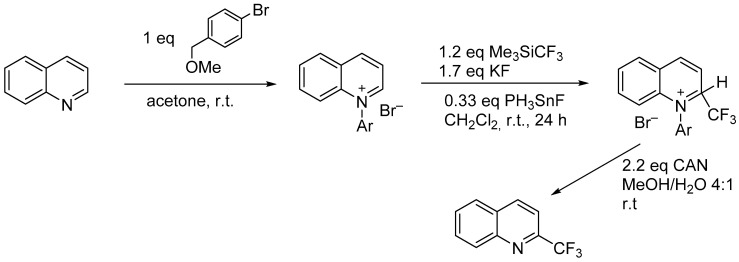
Trifluoromethylation of azines by nucleophilic aromatic substitution.

Although no specific examples have been reported in the literature, some reactions described in the literature for azines with up to three aromatic rings (phenanthridines) should be applicable to azahelicenes of a similar structure. One instance concerns the trifluoromethylation of azines through the formation of their azinium quaternary salts, with a more pronounced electrophilicity [35]. The azine is converted into its N-p-methoxybenzyl azinium bromide, which is then reacted with trifluoromethyl carbanions, generated by Me_3_SiCF_3_ in the presence of the fluoride anion. The resulting dihydroazine is then oxidatively deprotected and converted into the trifluoromethylated azine (Scheme 21).

In all cases, the CF_3_^−^ anion is reported to add exclusively to the positions 2 and 6 of the azinium ring, which is a regioselectivity typical of hard nucleophiles. For quinoline, the overall yield of the three steps is 55%.

### 3.3. The Minisci Reaction

One of the simplest and most effective ways to introduce a substituent into the activated ortho- and para-positions of azine rings is nucleophilic radical substitution, known as the Minisci reaction [36]. In general, the reaction is performed by generating a nucleophilic carbon-centered free-radical, such as alkyl, acyl, aryl, and benzyl radicals, in the presence of a protonated heteroaromatic base and a suitable oxidant for the rearomatization of the substrate. In this way, it is possible to attach alkyl chains of variable lengths to the azahelicene, bearing a variety of functional groups at the extremity, such as –OH, double bonds, acyl groups, halogens, and more. Following this strategy, it is possible to bind a functional group to a helicene structure, whose distance from the aromatic core can be modulated by inserting an appropriate spacer chain.

**Figure 4:**
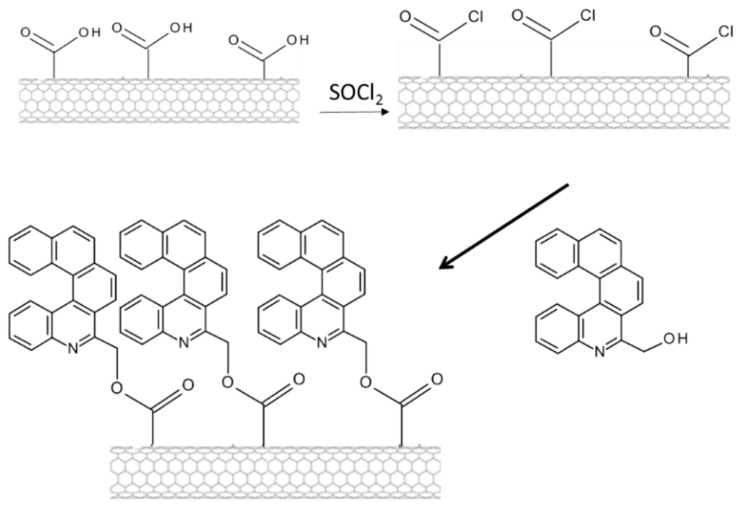
Functionalization of carbon nanotubes by 6-hydroxymethyl-5-aza[5]helicene.

Nucleophilic carbon-centered radicals are very reactive and selective species, which can attack aromatic systems with the opposite reactivity and selectivity compared to the Friedel–Crafts reaction. Carbon-centered radicals have a nucleophilic character if the carbon atom adjacent to the one bearing the radical does not bear an electron-withdrawing group. Such radicals can be obtained by hydrogen atom abstraction from alcohols, ethers, aldehydes, or hydrocarbons by oxygen-centered radicals, generated by peroxides, hydroperoxides, or dioxiranes [37,38], by the oxidative decarboxylation of carboxylic acids by the persulfate/Ag^+^ system [39] or by halogen atom abstraction by C- [40], Si- [41], or Sn-centered radicals [42] from alkyl halides. If a nucleophilic radical is generated in the presence of a protonated azine, the latter will act as a radical trap, undergoing radical attack at the ortho- and para-positions to the nitrogen atom. Rearomatization can be performed by the same radical-generating system or by oxidising the species present in the reaction medium. This approach was used to introduce 5-aza[5]helicene onto a –CH_2_OH substituent, in the position adjacent to the nitrogen atom [5]. The –CH_2_OH group was introduced using a Fenton radical-generating system (H_2_O_2_/Fe^2+^) in CH_3_OH as a solvent, in the presence of 5-aza[5]helicene protonated by an equimolar amount of CF_3_COOH. This azahelicene has only one activated position adjacent to the nitrogen atom and therefore can only give rise to the formation of the six-substituted derivative. The –OH group of 6-hydroxymethyl-5-aza[5]helicene was then reacted with the COOH group of functionalized carbon nanotubes to obtain a sensor for water vapor (Figure 4) [5].

**Scheme 22:**
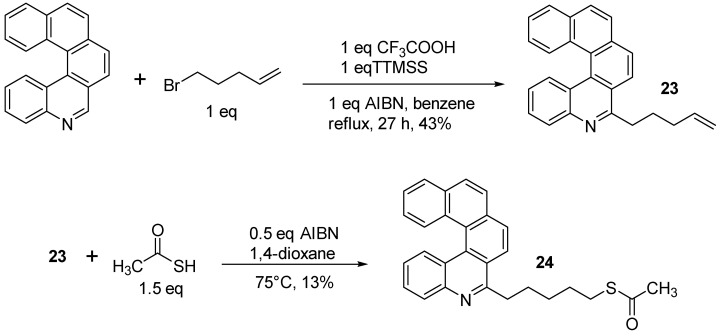
The formation of S-[5-(6-(5-aza[5]helicenyl)pentyl] ethanethioate **24**.

A similar approach, on the same aza[5]helicene, used 5-bromo-1-pentene as the radical precursor and the TTMSS/AIBN system to generate silicon radicals for the abstraction of bromine atom [43] to produce a pentenyl radical. In this way, the 5-pentenyl-substituted azahelicene **23** is obtained, and the double bond can be reacted to introduce different types of further functional groups. In particular, in [44], it was reacted with thioacetic acid CH_3_CS-OH, to obtain S-[5-(6-(5-aza[5]helicenyl)pentyl] ethanethioate **24** (Scheme 22).

Ideally, the double bond could be reacted in several different ways to introduce other functional groups, depending on the scope of the procedure. The thioester in this case was introduced as a thiol precursor, and the acetyl was removed in a basic medium to attach the organic moiety to a nanostructured gold surface.

## 4. Positively Charged Azahelicenes

Azahelicenes can be either neutral or charged molecules. Most of the described azahelicenes have no electrical charge. However, if there is a charge on the helicene structure, it should be balanced by the presence of a counterion. This counterion can affect the physical properties of the molecule, and it can also have a chiral structure.

Azahelicenes can bear a positive charge, which can be originated either by quaternarization of the nitrogen atom or by a molecular framework in which a stable carbocation is embedded.

### 4.1. Quaternary Azahelicenium Salts

Azahelicenes are basic molecules [8,9], which can be protonated by acids and also act as nucleophiles towards reactants such as alkyl halides, turning into the corresponding N-alkylated quaternary salts. The synthesis and characterization of N-methyl-5-aza[5]helicenium iodide is reported in [8]. It was obtained by dissolving 5-aza[5]helicene in CCl_4_, then by treating it with 10 equiv. of CH_3_I. The quaternary salt precipitates and is collected by filtration. It is also possible to obtain these quaternary salts by filtration upon dissolving the azahelicene in any neat liquid alkyl iodide. These salts are much more soluble in polar solvents than the parent azahelicenes, and N-methyl-5-aza[5]helicenium iodide is rather water-soluble. This salt was found to bind effectively to DNA [45] and the binding parameters were found to be strongly dependent on the nature of the counterion. Actually, any N-alkylhelicenium iodide can undergo counterion exchange simply by dissolving it in water and adding an equimolar solution of the silver salt of the anion of interest: AgI will precipitate and can be removed by filtration. To obtain the azahelicenium sulfate, on the other hand, it is sufficient to use (CH_3_)_2_SO_4_ as alkylating agent. The emission properties of these salts were studied to use them for cell labeling [46].

It was observed that in the presence of two nitrogen atoms in a diazahelicene, the alkylation of one of them hinders quaternarization on the other, as the positive charge developed on it exerts a strong inductive effect over the second one, whose nucleophilicity is consequently reduced [47]. Thus, the second alkylation to give the doubly quaternarized derivative is more difficult than the first one, and requires harsher experimental conditions. The consequence is that it is possible to selectively accede to both mono- and di-substituted salts. However, when the two nitrogen atoms are heterotopic, the question arises about which one would be alkylated first. In [47], the question was addressed by DFT calculations, only to verify that the very small difference in electron density among the two nitrogen atoms was not significant enough to justify the experimentally observed preferential alkylation of the nitrogen in position 2 with respect to the one in position 12.

**Figure 5:**
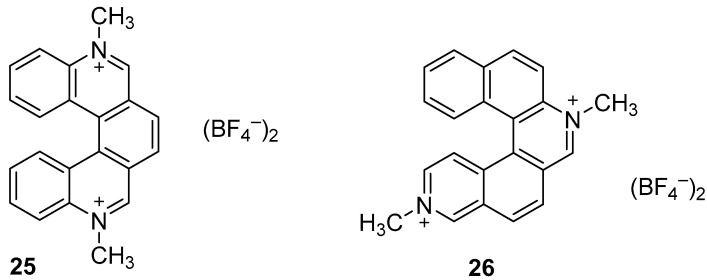
Two viologen-like dications.

Viologens are the diquaternary salts of 4,4′-bipyridine; in the quest for π-extended helical viologen, 5,10-dimethyl-5,10-diaza[5]helicene bis-tetrafluoroborate **25** and 3,8-dimethyl-3,8-diaza[5]helicene bis-tetrafluoroborate **26** (Figure 5) were synthesized by the treatment of the parent diaza[5]helicene with 2 equiv. of trimethyloxonium tetrafluoroborate in dichloromethane under nitrogen for several hours. The yields were 73% for **25** and 70% for **26**. [48]. These products were characterized with respect to their photophysical and photochemical behavior; the proposed applications included molecular machines and host–guest inclusion compounds.

The quaternarization of nitrogen was also realized to bind azahelicene moieties to structures such as cyclodextrins [49]. In particular, 3-aza[6]helicene was reacted with 3 equiv. of 6-deoxy-6-iodo-β-CD in DMF at 90 °C under nitrogen for 2 days, then the mixture was poured in cool acetone, where the product precipitated, was collected by filtration, and was purified by column chromatography, obtaining a 57% yield.

### 4.2. Helquats and Azoniahelicenes

Helquats and Azoniahelicenes are helicene-like compounds in which the nitrogen atom is located at the junction between two rings, so it bears a positive charge. Helquats are only partially aromatic, while azoniahelicenes are fully aromatic molecules.

**Scheme 23:**
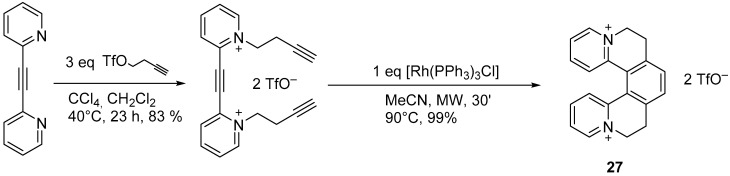
Synthesis of a [5]helquat.

Helquats can be prepared relatively easily through the [2 + 2 + 2] cycloisomerization approach. Helquat dyes combine a cationic hemicyanine with a helicene-like motif to form a new blueprint for chiral systems with large and tunable nonlinear optical (NLO) properties. Not many examples have been described in the literature. The first synthesis, reported in [50], yielded helquat (**27**) up to gram scale (Scheme 23).

**Scheme 24:**
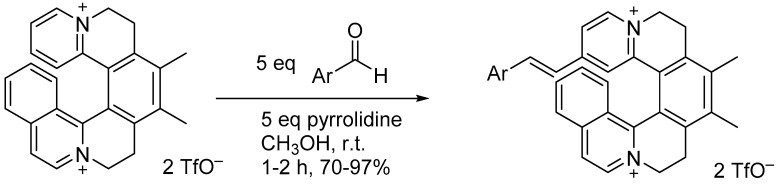
Knoevenagel condensation to substituted helquats.

By using the appropriate precursors, [5]helquats similar to **27** bearing methyl substituents in various positions, as well as [6]- and [7]-helquats, were prepared [51,52,53]. Such structures can be functionalized in the para-position to the nitrogen atom by means of Knoevenagel condensation [51], as reported in Scheme 24.

Aryl groups can bear electron-donating substituents, such as amino or methoxy groups [52,53].

**Scheme 25:**
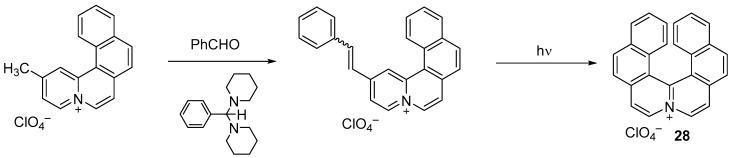
Synthetic strategy to obtain azoniahelicene **28**.

Even sparser literature is available in English about azoniahelicenes. The azonia derivative of hexahelicene **28**, 4a-azoniaphenanthro[3,4-c]phenanthrene perchlorate, was first synthesized by photocyclization of 2-styryl naphtho[1,2-a]quinolizinium perchlorate [54] (Scheme 25).

**Scheme 26:**
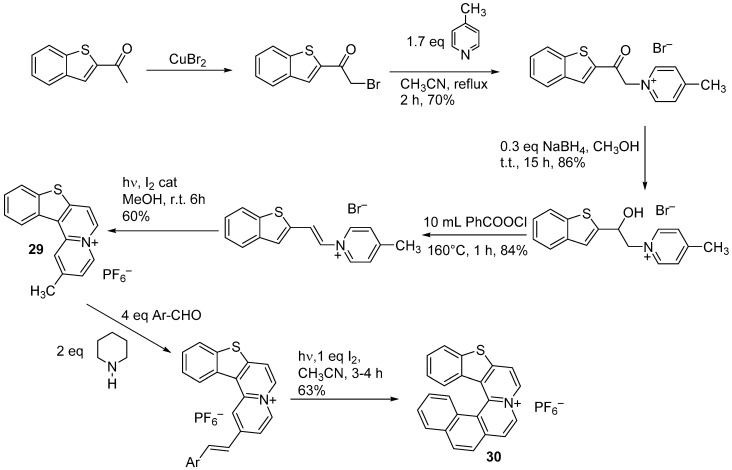
Synthesis of azoniathiahelicene **30**.

A synthesis of azoniathiahelicenes was obtained through the strategy shown in Scheme 26 [55], involving the preparation of 2-methylbenzothieno[3,2-a]quinolizinium hexafluorophosphate **29** and its Knoevenagel condensation with benzaldehyde. Photocyclization of the obtained precursor yields azoniahelicene **30**. Just like in other instances involving the photocyclization of styryl intermediates, by using an appropriately substituted aldehyde, a substituted azoniahelicene could be obtained.

### 4.3. Azahelicenes with Cationic Framework

**Scheme 27:**
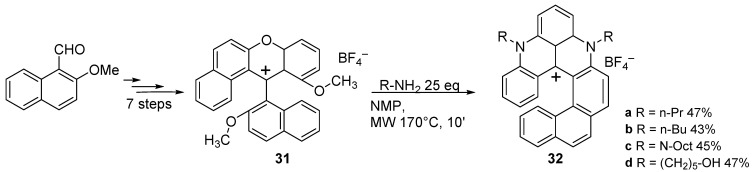
Formation of positively charged diaza[6]helicene **32**.

Cationic azahelicenes, where a carbon atom in the molecular framework bears a positive charge, are both very stable as carbocations and are also configurationally stable. They can be prepared following a relatively simple synthetic strategy and then functionalized onto the aromatic ring in diverse ways. In one instance [56], the synthesis starts from 2-methoxy-1-naphthaldehyde and, through seven steps, brings about the intermediate formation of 10-methoxy-11-(2-methoxy-1-naphthyl)-11H-benzo[a]xanthenium carbocation **31**, which is then heated with the appropriate aliphatic amine under microwave irradiation to yield the blue salts **32a**–**d** in a moderate yield (Scheme 27).

**Scheme 28:**
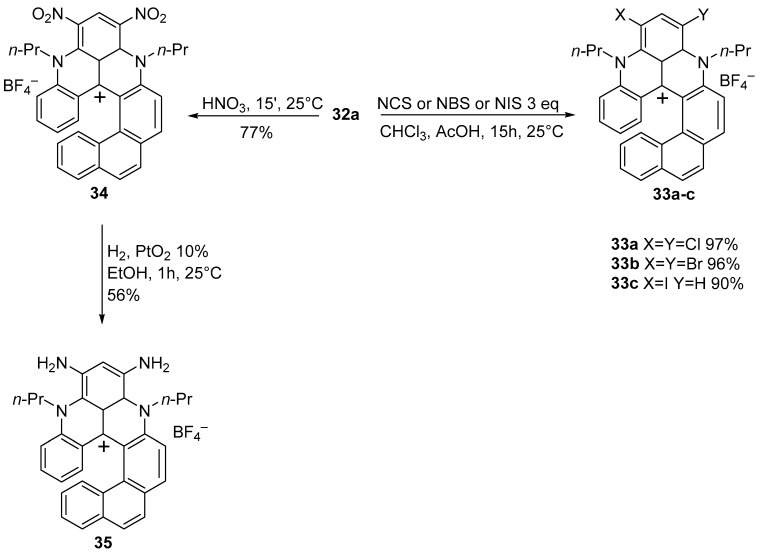
Introduction of nitro and amino groups and of halogen atoms on the azahelicene scaffold.

Product **32a** can undergo further transformations to yield a series of disubstituted halogen or nitrogen-containing products **33a**–**c**, **34**, and **35** (Scheme 28).

**Scheme 29:**
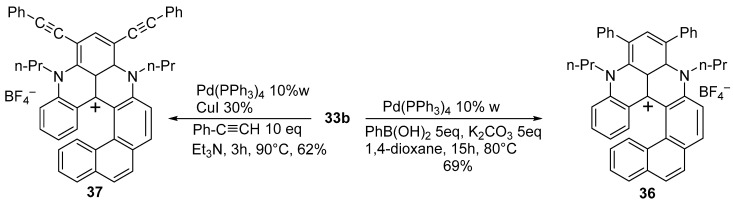
Introduction of aryl and alkynyl groups by the substitution of bromine.

The brominated product **33b** can be further reacted under Suzuki–Miyaura and Sonogashira conditions to yield the diphenyl (**36**) and diphenylethynyl (**37**) derivatives (Scheme 29).

**Scheme 30:**
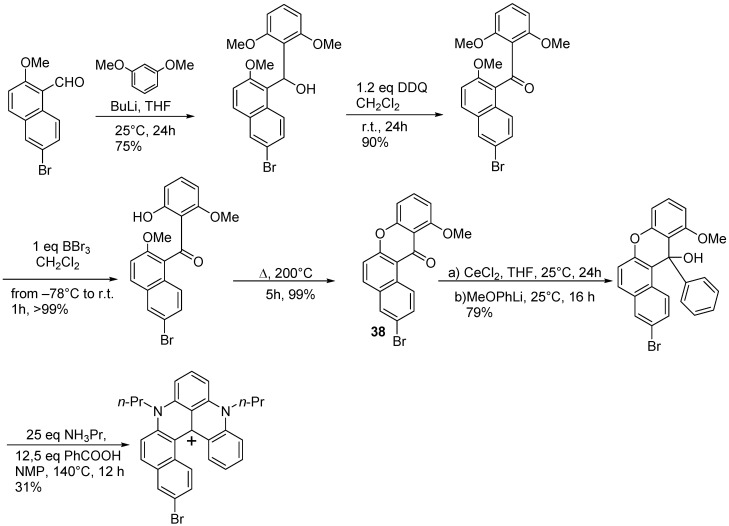
Introduction of aryl and alkynyl groups by the substitution of bromine.

A very recent paper [57] shows the preparation of a cationic Br-substituted diaza[5]helicene, as depicted in Scheme 30.

**Figure 6:**
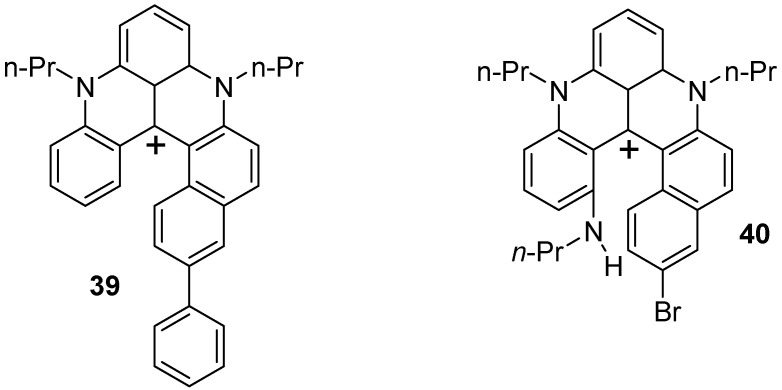
Cationic diazahelicene structures.

Intermediate **38** can be prepared on a multigram scale and can also be used for the preparation of structurally similar dioxa- and azaoxa-helicenes. It is also possible to prepare the related compounds **39** and **40** (Figure 6) through the transformation of dioxaderivatives.

Compound **39** shows remarkable emitting properties, with a maximum at 624 nm and a quantum yield of 0.42, with a value considered high for a red-emitting helicene.

## 5. Conclusions

Six-membered ring azahelicenes are an interesting family of compounds, on which the literature is still relatively sparse, particularly concerning substituted terms. The synthetic approach to substituted azahelicenes can be either to synthesize a helicenic framework already containing the desired functionality, or to add the wanted functional group to the previously prepared azahelicene, by taking advantage of the presence of the nitrogen atom(s), which can be used to direct transformations selectively to the desired positions on the aromatic framework. Several methods have been reported to transform easily introduced functional groups, such as halogen atoms, to other, more useful groups, such as amino, hydroxyl, or unsaturated functionalities. The introduction of functional groups has been exploited to attach azahelicene moieties to several kinds of structures, ranging from cyclodextrins and carbon nanotubes to metal surfaces. Heteroatom-containing functional groups can furthermore be useful for metal ion complexation.

The nitrogen atom itself can be quaternarized to yield azahelicenium salts, which are one of several kinds of charged compounds based on the azahelicene framework, among which helquats, azoniahelicenes, and azahelicenes with a cationic framework are discussed. Access to all these compounds may open the way to many and diverse applications of azahelicenes, thus allowing for the exploitation of their remarkable electronic and photophysical properties.
